# Time to Death and Donation After Circulatory Death Kidney Transplant Outcomes: Opportunities for Improved Utilization in the United States

**DOI:** 10.1111/ctr.70548

**Published:** 2026-04-24

**Authors:** Dharesh Raj Amarnath, Tanissha Sanjay Raj Kalpana, Georgios Kourounis, Abdullah K. Malik, Jennifer Philip, Emily R. Thompson, Emily Glover, Gavin J. Pettigrew, Chris Callaghan, Neil Sheerin, Colin Wilson, Samuel J. Tingle

**Affiliations:** ^1^ Newcastle University School of Medicine Newcastle upon Tyne UK; ^2^ NIHR Blood and Transplant Research Unit, Newcastle University and Cambridge University Newcastle upon Tyne UK; ^3^ Institute of Transplantation The Freeman Hospital Newcastle upon Tyne UK; ^4^ Devarajan Medical Center Arani Tamil Nadu India; ^5^ Translational and Clinical Research Institute Newcastle University Newcastle upon Tyne UK; ^6^ Division of Transplantation University of Wisconsin School of Medicine and Public Health Madison Wisconsin USA; ^7^ Renal Services, Newcastle Upon Tyne Hospitals NHS Foundation Trust Newcastle upon Tyne UK; ^8^ Department of Surgery University of Cambridge Cambridge UK; ^9^ Department of Nephrology and Transplantation Guy's Hospital, Guy's and St Thomas’ NHS Foundation Trust London UK

**Keywords:** donation after circulatory death (DCD), donor utilization, graft survival, kidney transplantation, machine perfusion, normothermic regional perfusion, time to death

## Abstract

**Background:**

Concerns persist that prolonged time to death (TTD) following withdrawal of life‐sustaining treatment may impair organ quality. Although previous European studies have demonstrated that prolonged TTD does not impact post‐transplant kidney graft survival, it remains unclear whether these findings apply to the US donor pool.

**Methods:**

We used OPTN data on adult DCD single‐kidney transplants (2010–2023). Multiple imputation was used for missing data. Multivariable regression models, with restricted cubic splines for non‐linear modelling, were used to evaluate the impact of donor TTD on kidney transplant outcomes.

**Results:**

Median donor TTD was 14 min (IQR, 10–21 min). Donor TTD was not associated with recipient graft survival (*p* = 0.469), mortality (*p* = 0.528) or 1‐year eGFR (*p* = 0.393). These findings were consistent regardless of normothermic regional perfusion use (NRP cohort: *n* = 1227; non‐NRP cohort: *n* = 35 328), and within the large ex‐situ hypothermic machine perfusion cohort (HMP‐ cohort: *n* = 22 218). Only 4.1% of transplanted DCD kidneys were from donors with TTD of over 60 min, and just 0.1% exceeded 120 min.

**Conclusions:**

Increasing donor TTD was not associated with worse post‐kidney‐transplant outcomes within this cohort predominantly comprising donors with TTD 0–60 min. In contrast to this US setting, a previous UK study reported that a higher proportion of transplanted DCD kidneys were from prolonged TTD donors (12.3% from donors TTD >60 min; 4.2% from >120 min). This highlights an opportunity to safely expand the US DCD donor pool, especially in the era of machine perfusion where viability assessment may provide an additional safeguard.

## 1 Introduction

The survival benefit of kidney transplantation in the management of patients with end‐stage kidney disease (ESKD) has been well established during the past five decades [1]. Nevertheless, the rising prevalence of ESKD has resulted in a significant shortage of kidneys for transplantation. The adult kidney transplant waiting list in the United States continues to expand, with 46 661 new candidates added in 2023, surpassing the previous peak of 42 933 in 2019 [2]. This growing demand, combined with a persistent gap between organ availability and the number of patients needing transplants, highlights the urgent need to improve donor organ utilization and maximize the number of kidneys appropriately used for transplantation.

One strategy to optimize organ use is to challenge misconceptions that currently limit kidney utilization. Following withdrawal of life‐sustaining treatment (WLST) in potential donation after circulatory death (DCD) donors, donor warm ischemic time (DWIT) can be split into time to death (TTD; withdrawal of treatment to asystole) followed by asystolic time (from asystole to abdominal aortic cold flush). During TTD, there is some level of blood flow to donor kidneys, whereas asystolic time represents a period of absent blood flow. We have previously identified that these physiologically distinct time periods do have differential impacts on clinical outcomes and should be analyzed separately rather than grouping them as DWIT [3, 4, 5, 6].

Although we have demonstrated a lack of impact of kidney TTD on post‐transplant outcomes in the UK cohort, it is important to note that DCD populations differ substantially between the UK and the US (such as differing donor age and cause of death distributions) [3]. Whilst UK retrieval services are standardized, Organ Procurement Organizations in the US each have different DCD protocols and withdrawal/retrieval practices. There are also differences regarding the use of perfusion technologies, with the US using ex situ hypothermic machine‐perfusion (HMP) more frequently [3].

To address this knowledge gap, we evaluated the impact of donor TTD on US DCD donor kidney graft outcomes.

## 2 Methods

### 2.1 Setting

This population cohort study was performed using data collected from the Organ Procurement and Transplantation Network (OPTN) registry, specifically the standard transplant analysis and research (STAR) and Donor Network (DONORNET) supplement files. All data were provided in a fully anonymized format. Our Institutional Review Board equivalent determined that study‐specific ethical review, approval, or informed consent were not required. We included adult recipients (aged 18 years and over at the time of transplant) of single kidney‐only transplants performed between January 1, 2010, and September 30, 2023. Exclusion criteria were dual/multi‐organ/multi‐visceral and SPK transplants, retransplants, uncontrolled DCD donors, and missing data for TTD. We also excluded donors with missing asystolic time, as this was used to define NRP status. Data were extracted on September 30, 2024, which was the common closure date of the study, ensuring all patients had a minimum of 1‐year post‐transplant follow‐up period.

### 2.2 Outcomes and Definitions

The primary outcome was 1‐year graft survival, which is defined in the OPTN registry as graft loss (this includes all graft losses in the first year, including early graft losses) or death. Secondary outcomes were 1‐year patient survival (mortality), incidence of delayed graft function (DGF; defined as the need for dialysis in the first week after transplant), 1‐year estimated glomerular filtration rate (eGFR, using CKD‐EPI 2021 formula [7]), with recipients losing their graft before 1 year given a nominal eGFR value of 10 mL/min/1.73 m [2, 3] and 5‐year graft survival. A sensitivity analysis was performed using graft function at last follow‐up to assess death‐censored 1‐year graft survival. Median follow‐up was estimated using the reverse Kaplan–Meier method with graft survival [8].

TTD was defined as the time from WLST to donor asystole. Asystolic time was defined as the time from donor asystole to abdominal aorta cold flush. Functional time to death (FTTD) was the time from donor systolic blood pressure falling below 50 mm Hg until abdominal aorta cold flush. This definition aligns with commonly used approaches to functional warm ischemic time and accounts for variability in hemodynamic decline following WLST [3].

### 2.3 Cohorts for Analysis

To assess the impact of TTD on recipient outcomes, we included recipients of kidneys recovered through super‐rapid recovery (SRR) in the main cohort. Recipients of kidney recovered using normothermic regional perfusion were included as a separate NRP cohort, recognizing this as a novel technique and of specific interest. We also felt that any assumption about consistent impact of donor and recipient factors between SRR and NRP cohorts would not be valid. As procurement technique (SRR or NRP) is not discretely captured by OPTN, donor asystolic time (asystole to cold flush, which signifies the end of NRP in NRP cases) was used to define procurement technique. Donors with asystolic time of over 30 min were defined as NRP; those with asystolic time of less than 30 min were defined as SRR. This approach to differentiate between SRR and NRP procurements has been used and validated previously [9, 10, 11].

### 2.4 Statistical Analysis

To account for missing data, multiple imputation was performed (aregImpute; Hmisc R package) to generate 20 imputed datasets [12]. This approach for multiple imputation uses predictive mean matching with bootstraps to build rich additive restricted cubic spline models [13, 14]. This was chosen in preference to multiple imputation by chained equations, as it preserves non‐linear relationships. For survival outcomes (graft survival, patient survival), we included the event indicator variable and the cumulative hazard of the event in the multiple imputation model to preserve relationships between outcome and missing covariates [15].

Multivariable Cox regression was performed to assess the impact of TTD on recipient graft survival and patient survival. In addition, multivariable logistic regression was performed to assess the impact on DGF. Results were pooled from 20 imputed datasets, adjusting for variance based on both within‐ and between‐imputation variation [16].

Adjustment for a wide range of confounders was performed. Potential confounders were selected based on previous research and clinical expertise; statistical variable selection techniques (e.g., stepwise selection) were avoided [17]. We employed hierarchical modelling to adjust for transplant center as a determinant of post‐transplant outcome; this was done using a frailty Cox model allowing transplant center to be modelled as a random effect.

Restricted cubic splines with four knots (fifth, 35th, 65th, and 95th percentiles) were used to analyze continuous variables known to have a strong correlation with the outcome or likely to have non‐linear relationships. An a priori decision was made to use splines for these variables, to avoid assumptions of linearity. For continuous variables not modelled with splines, those with significant right skew on visual assessment of histograms were Log2‐transformed. For such variables the effect estimates therefore relate to the predictor doubling in value.

Additional models were built which included interaction terms, as the impact of TTD on outcome may have differed with certain donor and transplant factors. Sensitivity analyses were also performed adjusting for additional potential confounders that were not included in the main models due to issues with missing data or multicollinearity [18].

Kaplan–Meier plots were generated to show crude graft and patient survival, stratified by TTD. Continuous variables are given as median and interquartile range. Outputs of models are given as effect estimates with 95% confidence intervals. All analyses were performed in R (R Foundation for Statistical Computing, Vienna, Austria) [18], using the following packages; tidyverse, rms, rmsMD, Hmisc and survminer [11, 12, 13, 19, 20, 21, 22]. rmsMD was used to generate the regression model tables and spline plots in the figures [20].

## 3 Results

**FIGURE 1:**
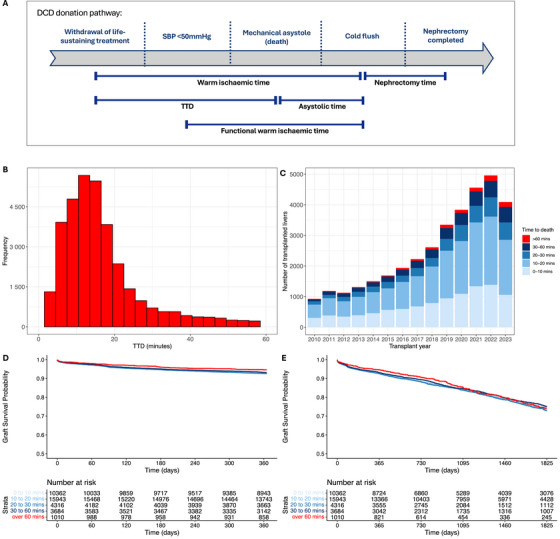
DCD donation pathway, cohort demographics and unadjusted graft survival. (A) Process of donation after circulatory death illustrating the definitions of TTD and asystolic time. (B) Number of kidney transplants over time based on TTD. (C) Number of kidneys transplanted based on TTD. (D) Above—Kaplan‐Meier curve of 1‐year graft survival in the SRR cohort stratified by TTD. Below—Number at risk over the 15‐year period. (E) Above—Kaplan‐Meier curve of 5‐year graft survival in the SRR cohort stratified by TTD. Below—Number at risk over the 5‐year period.

Between January first, 2010, and September 30th, 2023, we included 35 328 DCD kidney transplant recipients in the SRR cohort and 1227 in the NRP cohort. Information on cohort selection is given in our study flow diagram (Figure S1). Key cohort demographics are given in Table 1 (for SRR and NRP cohorts). Additional demographics and a full description of missing data are in Table S1. A diagram representing the DCD donation process is provided in Figure 1A. The median TTD was 14 min (IQR, 10–21 min), with the distribution shown in Figure 1B. Only 4.1% of transplanted kidneys had a TTD >60 min and 0.1% exceeded 120 min (Figure 1C). Crude 1‐ and 5‐year graft survival data stratified by donor TTD are shown as Kaplan–Meier plots (Figure 1D–E). Unadjusted graft survival was numerically similar with increasing TTD.

**TABLE 1:** Super‐rapid recovery (SRR) and Normothermic Regional Perfusion (NRP) cohort demographics.

Variable	SRR (*N* = 35 328)	NRP (*N* = 1227)	Overall (*N* = 36 555)
Donor TTD			
Median [Q1, Q3]	14.0 [10.0, 21.0]	13.0 [7.00, 19.0]	14.0 [9.00, 21.0]
**Donor functional TTD**			
Median [Q1, Q3]	2.00 [0, 5.00]	1.00 [0, 3.00]	2.00 [0, 5.00]
**Asystolic time**			
Median [Q1, Q3]	10.0 [8.00, 13.0]	−	−
Missing	14 506 (41.1%)	−	−
**Donor age (years)**			
Median [Q1, Q3]	42.0 [30.0, 52.0]	37.0 [27.0, 48.0]	42.0 [30.0, 52.0]
**Donor sex**			
Female	11 715 (33.2%)	284 (23.1%)	11 999 (32.8%)
Male	23 613 (66.8%)	943 (76.9%)	24 556 (67.2%)
**Recipient delayed graft function incidence**			
Yes	15 358 (43.5%)	356 (29.0%)	15 714 (43.0%)
No	19 967(56.5%)	871 (80.0%)	20 838 (57.0%)
Missing	3 (∼0%)	0 (0%)	3 (∼0%)
**Recipient age (years)**			
Median [Q1, Q3]	57.0 [47.0, 65.0]	53.0 [42.0, 63.0]	57.0 [47.0, 65.0]
**Recipient sex**			
Female	13 500 (38.2%)	496 (40.4%)	13 996 (38.3%)
Male	21 828 (61.8%)	731 (59.6%)	22 559 (61.7%)
**Recipient BMI (kg/m^2^)**			
Median [Q1, Q3]	28.5 [24.8, 32.6]	28.3 [24.4, 32.6]	28.5 [24.8, 32.6]
Missing	19 (0.1%)	0 (0%)	19 (0.1%)
**Cold ischemic time (h)**			
Median [Q1, Q3]	19.5 [15.0, 24.0]	19.6 [15.3, 24.0]	19.5 [15.1, 24.0]
Missing	135 (0.4%)	1 (0.1%)	136 (0.4%)
**Machine perfusion use**			
SCS	7163 (20.3%)	270 (22.0%)	7433 (20.3%)
cHMP	12 324 (34.9%)	433 (35.3%)	12 757 (34.9%)
eHMP	2825 (8.0%)	89 (7.3%)	2914 (8.0%)
HMP	6339 (17.9%)	208 (17.0%)	6547 (17.9%)
Missing	6677 (18.9%)	227 (18.5%)	6904 (18.9%)

Abbreviations: BMI = body mass index; PDCA = pre‐donation cardiac arrest; TTD = time to death; FTTD = functional time to death; cHMP = Continuous Hypothermic Machine Perfusion; eHMP = End Hypothermic Machine Perfusion.

### 3.1 Impact of Donor TTD in the SRR Cohort

**FIGURE 2:**
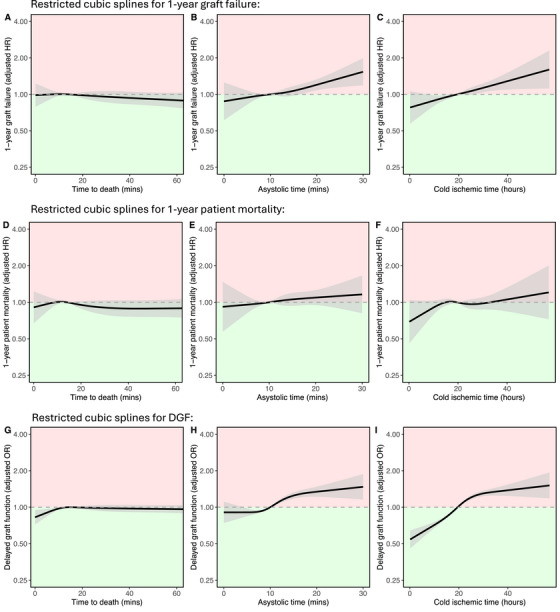
Impact of donor ischemic times on 1‐year graft failure, 1‐year patient mortality, delayed graft function using restricted cubic splines with 4 knots in the SRR cohort. Lines represent restricted cubic splines with grey shaded areas for 95% confidence intervals. Associations between (A) TTD, (B) asystolic time, and (C) cold ischemic time against 1‐year graft failure (derived from model in Table S2). Associations between (D) TTD, (E) asystolic time, and (F) cold ischemic time against 1 year patient mortality (derived from model in Table S3). Associations between (G) TTD, (H) asystolic time, and (I) cold ischemic time against delayed graft function (derived from model in Table S5). The green region represents superior outcomes compared to the reference, while the red region signifies inferior outcomes.

A multivariable Cox regression model was used to assess the association of TTD with recipient 1‐year graft survival, adjusting for a wide range of factors (Table S2, splines in Figures 2A–C and S2). Donor TTD was not significantly associated with 1‐year graft survival (*p* = 0.469; see spline in Figure 2A, model summary in Table S2).

We hypothesized that the impact of TTD may have differed based on other donor or transplant factors. This was analyzed by including interaction terms to the model in Table S2. We did not identify any differences in the impact of TTD on graft survival based on the following factors: asystolic time (interaction *p* = 0.869), CIT (interaction *p* = 0.586), and donor age (interaction *p* = 0.438).

Kidneys were preserved using ex situ HMP in 22 218 cases (60.8%). The impact of TTD also did not differ based on HMP use (interaction *p* = 0.831). Figure S3 shows that prolonged TTD was not significantly associated with graft survival even in the setting of HMP.

Donor TTD was not associated with 1‐year patient survival (*p* = 0.528; Figure 2D, Figure S4, Table S3) or 1‐year eGFR (*p* = 0.393, Figure S5, Table S4). TTD also had no clinically relevant impact on DGF rate, and there was no trend toward increasing DGF incidence with longer TTD values (Figure 2G, Figure S6, Table S5).

Additional multivariable Cox regression models were built to analyze the impact of TTD on longer term 5‐year graft and patient survival, adjusting for the same factors in Table S2 where TTD again had no impact on longer‐term outcomes (Tables S6 and S7, Figures S7 and S8).

A sensitivity analysis was performed using an identical model to Table S2 but with death‐censored graft survival censored at 1 year as the outcome. Results were consistent with the main analysis, and TTD was not associated with outcome (*p* = 0.166).

### 3.2 Accounting for Clustering

Sensitivity analysis was performed to account for clustering within different transplant centers. Adding transplant center as a random effect in a Cox frailty model was consistent with the main results. Although there was significant variation in graft survival by transplant center (*p *< 0.001, Chisq = 157.6, df = 81.8), there were no meaningful changes observed to the splines for the impact of any of the ischemic times on outcome.

Additionally, we adjusted the model variance for clustering based on a single donor donating two kidneys. This did not meaningfully change the main results; the association between TTD and outcome remained non‐significant.

### 3.3 Impact of Donor Functional TTD

Some suggest that functional time to death (FTTD) is a better metric for indicating the level of ischemic injury prior to death. We applied the same modelling strategy as described above to analyze the impact of FTTD on 1‐year graft survival. Donor FTTD was not significantly associated with graft survival (*p* = 0.248; Figure S9, Table S8).

### 3.4 Impact of Other Ischemic Times

In the multivariable models described above, increasing asystolic time was significantly associated with worse 1‐year graft survival (*p* < 0.001; Figure 2), lower 1‐year eGFR (*p* < 0.001; Figure S5) and higher rates of delayed graft function (*p* < 0.001; Figure 2). No significant associations were observed between asystolic time and 1‐year patient survival (*p* = 0.441; Figure 2) or with longer‐term outcomes including 5‐year graft survival (*p* = 0.074; Figure S7) and 5‐year patient survival (*p* = 0.091; Figure S8).

Cold ischemic time was significantly associated with worse 1‐year graft survival (*p* < 0.001; Figure 2) and increased delayed graft function (*p* < 0.001; Figure 2). Significant associations were also observed with 5‐year graft survival (*p* < 0.001; Figure S7) and 5‐year patient survival (*p* = 0.017; Figure S8), 1‐year eGFR (*p* < 0.001; Figure S5).

#### 3.4.1 NRP Cohort

**FIGURE 3:**
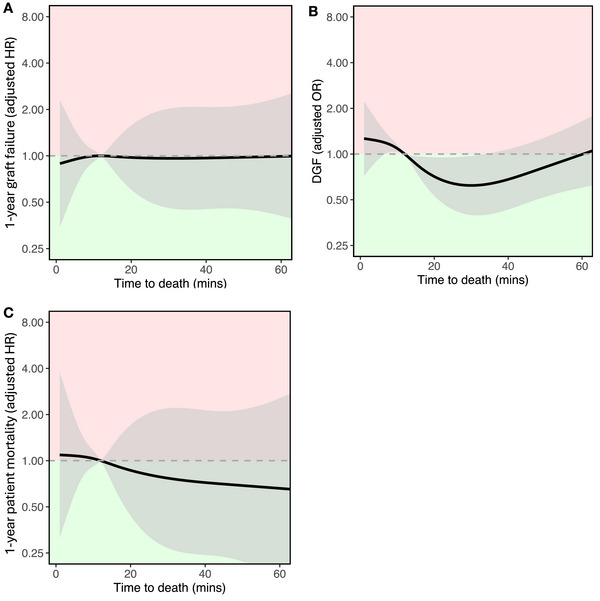
Association of TTD with (A) 1‐year graft failure, (B) DGF, (C) 1‐year patient mortality, derived from models adjusting for machine perfusion, cold ischemic time, donor age, year of transplant in the NRP cohort. Lines represent restricted cubic splines with grey shaded areas for 95% confidence intervals. The green region represents superior outcomes compared to the reference, while the red region signifies inferior outcomes.

A multivariable analysis was performed to assess the impact of TTD on 1‐year graft survival in the setting of NRP, adjusting for donor age, ex‐situ hypothermic machine perfusion, cold ischemic time and year of transplant. No association was observed between donor TTD and 1‐year graft survival in this cohort (*p* = 0.996; see splines in Figure 3A). The limited number of events (graft losses) prevented multivariable analyses with a greater number of confounders. Similarly, TTD was not associated with 1‐year patient survival (*p* = 0.890; see splines Figure 3B,C), 1‐year eGFR (*p* = 0.961), long term 5‐year graft (*p* = 0.612) and 5‐year patient survival (*p* = 0.498).

Although TTD was associated with DGF in this NRP cohort (*p* = 0.024; Figure 3B), donor TTD of 60 min did not result in a significantly higher incidence of DGF when compared with the median TTD of 13 min in the NRP cohort (OR 0.995, 95% CI 0.602–1.646).

We then applied the same modelling strategy as described above to analyze the impact of functional TTD (FTTD) on 1‐year graft survival with NRP use. Donor FTTD was not associated with graft survival (*p* = 0.165).

## 4 Discussion

This study is the largest analysis to date on the impact of donor time to death (TTD) on kidney transplant outcomes, expanding a literature base that has so far been drawn largely from smaller European cohorts [3, 5]. As shown in Table 1, the incidence of DGF was 43.5% in the SRR cohort and 29.0% in the NRP cohort, with an overall incidence of 43.0%. Our findings demonstrate that donor TTD is not significantly associated with adverse post‐transplant graft or patient outcomes, irrespective of the use of NRP or hypothermic machine perfusion. These findings are consistent with our previous UK study which demonstrated that kidneys from donors with TTD up to at least 3 h can be transplanted with good outcomes, supporting broader acceptance of prolonged TTD donors [3].

In this US study, 4.1% of transplanted DCD kidneys were from donors with TTD over 60 min and 0.1% exceeded 120 min. In comparison, in the UK 12.3% and 4.2% of transplanted DCD kidneys come from donors with TTD exceeding 60 and 120 min, respectively. In the US, if transplant of DCD donors with TTD <60 min remained constant, and transplant of kidneys from donors with TTD >60 min was expanded such that 12.3% of transplants were from such donors (matching the UK data), this would represent a 9.4% increase in DCD kidney transplants. Based on latest SRTR data of showing DCD kidney transplants approaching 10 000 per year, this would equate to 940 additional transplants yearly [23]. Whether this rough approximation is feasible depends on a range of factors, including availability of donors and transplantation logistics.

Transplant logistics, retrieval costs and operational burdens are critical factors in decision‐making around stand‐down time policies. Our findings demonstrate no inferior outcomes with prolonged TTD kidney grafts. Therefore, increased utilization of such organs by transplant centers would encourage OPOs to extend the stand‐down times to increase the number of transplants. In this setting of extending the stand‐down times, additional retrieval costs relate only to increasing wait times in the donor hospital (rather than costs associated with equipment or retrieval team transport). Previous studies have shown that many potential DCD donors proceed to circulatory death within 1–3 h of withdrawal of life‐sustaining treatment. In one study, although the median time to death was 36 min, 43.5% of donors were still alive at 1 h; of these, 29.0% proceeded to death within 3 h [24]. A US study reported a 9.8% increase in successful transplants, by routinely accepting donors with TTD of 1–2 h [25]. Our group has demonstrated that the minimum stand‐down time of 3 h in the UK achieved a 14.1% increase in DCD transplants versus a theoretical 1‐h stand down policy, without any negative impact on post‐transplant outcomes [3, 26, 27].

However, implementation of prolonged stand‐down time policies may be challenging. Many DCD donors in the US are managed in hospitals that are not transplant centers, where operating room availability (especially during peak hours) may limit the feasibility of extended waiting periods. In addition, DCD protocols are generally determined by local hospital administrations. Therefore, any changes to stand‐down policies may need to be reviewed and accepted by each individual hospital.

Ongoing research to predict which potential DCD donors will proceed to death within certain timeframes may further allow logistic optimization [25, 28]. Such prediction tools could enable both earlier stand‐down in ‘futile’ potential donors that are unlikely to proceed (with benefits for retrieval team morale), whilst still allowing safe utilization of organs from donors which are likely to take 60–180 min to proceed.

This study advances previous evidence by analyzing 36 555 US kidney recipients, a cohort more than five times larger than our earlier UK study [3], enabling robust 1‐ and 5‐year outcome assessment, sensitivity analyses, and evaluation of functional TTD (FTTD). Compared with the previous UK cohort, this US cohort included younger donors (median [IQR] age 55 [44–63] years in the UK vs. 42 [30–52] years in the US) and a higher proportion of donors whose cause of death was drug overdose. This study also includes a substantially larger NRP cohort (1227 vs. 453) and a markedly greater number of HMP‐preserved grafts (22 218 vs. 469) compared with our previous work, allowing the clearest demonstration to date that neither NRP nor HMP alters the relationship between TTD and transplant outcomes.

Similar findings in other abdominal organ transplants suggest a broader physiological tolerance to prolonged agonal phases. We have previously reported comparable findings in DCD liver transplantation using both the US and UK data [5, 6]. Pancreas transplantation from DCD donors in the US remains uncommon, limiting robust analysis [29], although analyses using the UK data by our group suggest similar patterns [4].

The increasing use of ex‐situ machine perfusion and normothermic regional perfusion (NRP) in the US has significantly expanded the DCD donor pool [9]. These technologies may provide a ‘safety net’ through viability assessment [30], and appear to improve utilization across thoracic and abdominal organs [9]. Our findings are highly relevant in the current landscape of NRP use. Similar observations have been reported in DCD heart transplantation, where longer agonal phases reduced utilization but did not adversely affect post‐transplant survival when organs were accepted [9, 10].

Emerging evidence across solid organ transplantation highlights that injury is primarily driven by the no‐flow asystolic phase rather than the preceding agonal period [5]. Most previous studies report composite warm ischemic time, which conflates physiologically distinct phases. Our findings reinforce the hypothesis that kidney graft injury is driven by the no‐flow asystolic period rather than TTD itself, strongly supporting separate reporting of these intervals. We provide further evidence that TTD and asystolic time have very different impacts on outcome, with asystolic time, but not TTD, causing clinically relevant graft injury. This further drives a push to avoid reporting undifferentiated WIT in future studies; instead, analyses should report TTD and asystolic time separately [3, 4, 5].

A key limitation of the STAR data used for this study is that only donors which proceeded to organ retrieval are included. We therefore have no data on donors where the retrieval team were stood down. This prevents us assessing the precise number of additional kidneys that could be made available in the US by extending stand‐down times. It also precludes assessment of which factors may predict whether a given potential DCD donor will proceed to donation. However, based on the UK data, the number of additional organs available for transplantation from the adoption of a minimum 3‐h wait policy in the US would be substantial. Additionally, the impact of asystolic time in the setting of NRP (time between asystole and initiation of NRP) could not be assessed, as this duration was not recorded by OPTN. Another limitation is selection bias, given the avoidance of prolonged stand‐down time in the US versus Europe. This is especially true for prolonged TTD over 120 min, as kidney transplants from such donors is currently a rare event in the US. However, to address selection bias, we adjusted for a wide range of potential confounders, which was possible due to the large cohort size, including all the critical factors known to impact post‐transplant outcomes. In addition, the finding that prolonged TTD does not negatively impact outcomes is consistent with UK data, where TTD up to 3 h are routinely accepted and donors with prolonged TTD are not a highly selected subgroup, with broadly similar donor characteristics across TTD strata.

### 4.1 Conclusion

Donor TTD does not appear to be a clinically significant predictor of post‐transplant outcomes in DCD kidney transplantation in this US cohort, and these results were consistent in the setting of NRP and ex‐situ HMP. Whilst the majority of donors had TTD values <60 min, we observed no trends of worsening outcomes beyond this time point, in line with previous studies from Europe. A smaller proportion of transplanted kidneys were from donors with TTD over 60 min in the US compared to the UK (4.1% in the US versus 12.3% in the UK), and very few exceeded 120 min (0.1% in the US versus 4.2 in the UK). Evidence for good outcomes from prolonged TTD donors is further supported by the UK data, where utilization of such donors is higher. Donors with prolonged TTD therefore represent an important cohort that should be prioritized by OPOs by extending stand‐down times (for example to a minimum 3‐h wait policy) to maximise recovery of organs from the donors which they are already attending. The expanding use of enhanced organ preservation techniques which improve graft outcomes including NRP and other machine perfusion techniques that enable viability assessment might provide additional safeguards that could be leveraged to increase safe acceptance and utilization of these organs.

## Author Contributions

D.R.A. had full access to all the data in the study and takes responsibility for the integrity of the data and the accuracy of the data analysis. Concept and design: S.T. Acquisition and cleaning of data: S.T., D.A., and T.S.R.K. Statistical analysis: S.T. and D.A. Interpretation of data: All authors. Drafting of the manuscript: T.S.R.K. and D.A. Critical review of the manuscript for important intellectual content: All authors.

## Funding

The authors declare that financial support was received for the research and/or publication of this article. S.J.T. was funded for this work via a Medical Research Council Clinical Research Training Fellowship (MRC/Y000676/1), which was part‐funded by Kidney Research UK. The work was supported by the National Institute for Health and Care Research (NIHR) Blood and Transplant Research Unit in Organ Donation and Transplantation (NIHR203332), a partnership between NHS Blood and Transplant, University of Cambridge and Newcastle University. The views expressed are those of the author(s) and not necessarily those of the NIHR, NHS Blood and Transplant or the Department of Health and Social Care.

## Conflicts of Interest

The authors have nothing to report.

## Supporting information




**Supplementary Materials**: ctr70548‐sup‐0001‐SuppMat.docx

## Data Availability

The data used in this manuscript were provided to us by OPTN. The raw data may be requested from OPTN through written request.
