# Supplementary material for: Time to Death and Donation After Circulatory Death Kidney Transplant Outcomes: Opportunities for Improved Utilization in the United States
Source: Clin Transplant. 2026 Apr 24;40:e70548. doi: 10.1111/ctr.70548 (PMC13108833; doi:10.1111/ctr.70548)
Supplement: Supplementary file 1 — Supplementary Materials: ctr70548‐sup‐0001‐SuppMat.docx [file CTR-40-e70548-s001.docx]

# Supplementary material


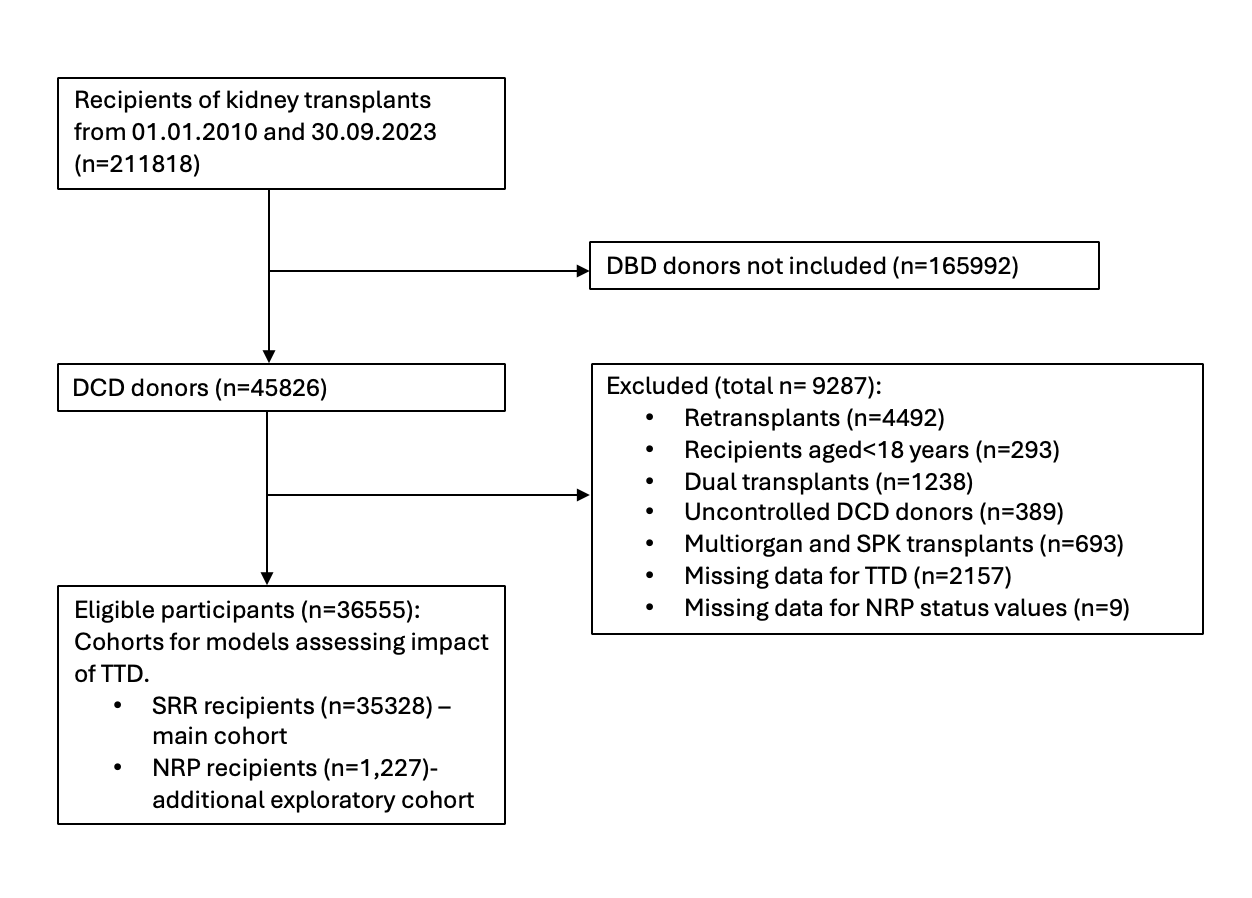


Supplementary figure 1:Study flowchart with inclusion and exclusion criteria

Supplementary Figure 2: Splines for 1-year graft survival using restricted cubic splines with 4 knots in the SRR cohort. Lines represent restricted cubic splines with grey shaded areas for 95% confidence intervals. Associations between (A) Donor age, (B) Donor BMI, (C) Donor peak albumin (D) Donor peak creatinine, (E) Donor admission to donation time, (F) Recipients age, (G) Recipient BMI, (H) Waitlist time, and (I) donor recipient weight ratio (J) Year of transplant (derived from model in Supplementary Table 2). The green region represents superior outcomes compared to the reference, while the red region signifies inferior outcomes.


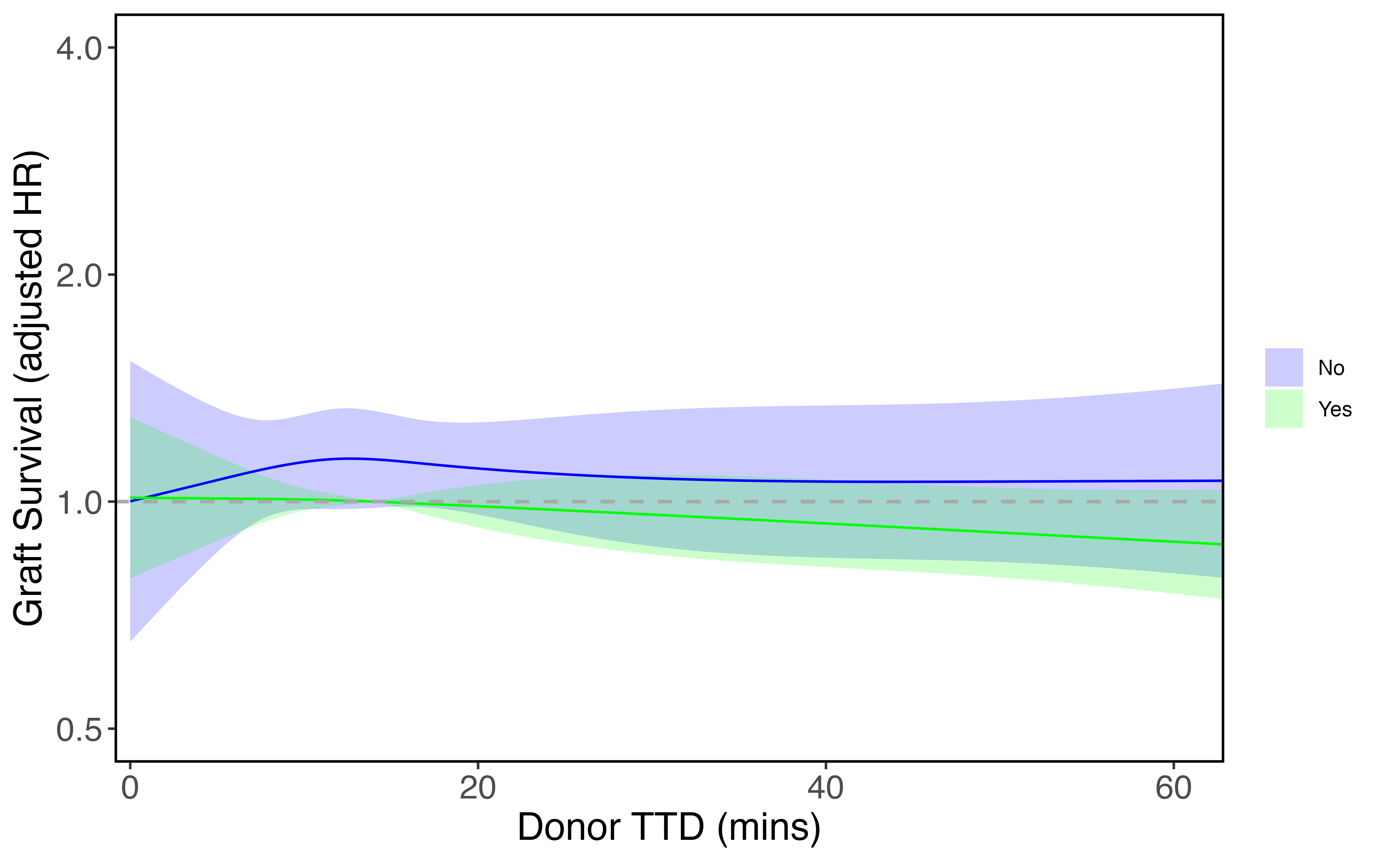


Supplementary Figure 3: Interaction plot between TTD and HMP in the SRR cohort.

**

Supplementary Figure 4: Splines for 1-year patient survival using restricted cubic splines with 4 knots in the SRR cohort. Lines represent restricted cubic splines with grey shaded areas for 95% confidence intervals. Associations between (A) Donor age, (B) Donor BMI, (C) Donor peak albumin, (D) Donor peak creatinine, (E) Donor admission to donation time, (F) Recipient age, (G) Recipient BMI, (H) Waitlist time, (I) donor recipient weight ratio and (J) Year of transplant (derived from model Supplementary Table 3). The green region represents superior outcome compared to the reference, while the red region signifies inferior outcome.

Supplementary Figure 5: Impact of donor ischemic times on 1-year eGFR, using restricted cubic splines with 4 knots in the SRR cohort. Association of (A) time to death, (B) asystolic time, and (C) cold ischemic time with 1-year eGFR. Lines represent the splines with the grey shaded area for 95% confidence interval.

Supplementary Figure 6: Splines for delayed graft function using restricted cubic splines with 4 knots in the SRR cohort. Lines represent restricted cubic splines with grey shaded areas for 95% confidence intervals. Associations between (A) Donor age, (B) Donor BMI, (C) Donor peak albumin, (D) Donor peak creatinine, (E) Donor admission to donation time, (F) Recipient age, (G) Recipient BMI, (H) Waitlist time, (I) donor recipient weight ratio and (J) Year of transplant (derived from model in Supplementary Table 5). The green region represents superior outcome compared to the reference, while the red region signifies inferior outcome.

Supplementary Figure 7: Splines for 5-year graft survival using restricted cubic splines with 4 knots in the SRR cohort. Lines represent restricted cubic splines with grey shaded areas for 95% confidence intervals. Associations between (A) time to death, (B )Asystolic time, (C) Donor age, (D) Donor BMI, (E) Donor peak albumin, (F) Donor peak creatinine, (G) Donor admission to donation time, (H) Recipient age, (I) Recipient BMI, (J) Waitlist time, (K) Cold ischemic time, (L) donor recipient weight ratio and (M) Year of transplant (derived from model in Supplementary Table 6). The green region represents superior outcome compared to the reference, while the red region signifies inferior outcome.

Supplementary Figure 8: Splines for 5-year patient survival using restricted cubic splines with 4 knots in the SRR cohort. Lines represent restricted cubic splines with grey shaded areas for 95% confidence intervals. Associations between (A) time to death, (B) Asystolic time, (C) Donor age, (D) Donor BMI, (E) Donor peak albumin, (F) Donor peak creatinine, (G) Donor admission to donation time, (H) Recipient age, (I) Recipient BMI, (J) Waitlist time, (K) Cold ischemic time, (L) donor recipient weight ratio and (M) Year of transplant (derived from model in Supplementary Table 7). The green region represents superior outcome compared to the reference, while the red region signifies inferior outcome.

Supplementary Figure 9: Impact of FTTD on 1-year graft survival using restricted cubic splines with 4 knots in the SRR cohort. Lines represent restricted cubic splines with grey shaded areas for 95% confidence intervals. Associations between (A) Functional time to death, (B) Asystolic time, (C) Donor age, (D) Donor BMI, (E) Donor peak albumin, (F) Donor peak creatinine, (G) Donor admission to donation time, (H) Recipient age, (I) Recipient BMI, (J) Waitlist time, (K) Cold ischemic time, (L) donor recipient weight ratio, (M) Year of transplant. The green region represents superior outcomes compared to the reference, while the red region signifies inferior outcomes.

Supplementary table 1: Full extended cohort demographics of the Super-rapid recovery (SRR) and Normothermic Regional Perfusion (NRP) demographics. Abbreviations: BMI=body mass index; PDCA= pre-donation cardiac arrest; TTD =time to death; FTTD= functional time to death; CHMP= Continuous Hypothermic Machine Perfusion; eHMP= End Hypothermic Machine Perfusion, BUN= Blood urea nitrogen, ATG= Anti-Thymocyte Globulin; HLA= Human Leukocyte Antigen; SCS= Static cold storage and CHMP= Continuous Hypothermic Machine Perfusion; eHMP= End Hypothermic Machine Perfusion

| **Variable** | **SRR (N=35328)** | **NRP (N=1227)** | **Overall (N=36555)** |
| --- | --- | --- | --- |
| **Donor TTD** |  |  |  |
| Median [Q1, Q3] | 14.0 [10.0,21.0] | 13.0 [7.00,19.0] | 14.0 [9.00,21.0] |
| **Donor functional TTD** |  |  |  |
| Median [Q1, Q3] | 2.00 [0,5.00] | 1.00 [0,3.00] | 2.00 [0,5.00] |
| **Asystolic time** |  |  |  |
| Median [Q1, Q3] | 10.0 [8.00,13.0] | - | - |
| Missing | 14506 (41.1%) | - | - |
| **Donor Age (years)** |  |  |  |
| Median [Q1,Q3] | 42.0 [30.0,52.0] | 37.0 [27.0,48.0] | 42.0 [30.0,52.0] |
| **Donor Sex** |  |  |  |
| Female | 11715 (33.2%) | 284 (23.1%) | 11999 (32.8%) |
| Male | 23613 (66.8%) | 943 (76.9%) | 24556 (67.2%) |
| **Donor BMI (kg/m²)** |  |  |  |
| Median [Q1,Q3] | 27.7 [23.8,32.6] | 27.1 [23.4,31.7] | 27.7 [23.7,32.6] |
| Missing | 757 (2.1%) | 21 (1.7%) | 778 (2.1%) |
| **Donor Ethnicity** |  |  |  |
| White | 28071 (79.5%) | 955 (77.8%) | 29026 (79.4%) |
| Asian | 648 (1.8%) | 12 (1.0%) | 660 (1.8%) |
| Black | 2867 (8.1%) | 89 (7.3%) | 2956 (8.1%) |
| Hispanic | 3331 (9.4%) | 149 (12.1%) | 3480 (9.5%) |
| Other | 411 (1.2%) | 22 (1.8%) | 433 (1.2%) |
| **Cause of Death** |  |  |  |
| Cerebrovascular/stroke | 5920 (16.8%) | 141 (11.5%) | 6061 (16.6%) |
| Anoxia with PDCA | 10182 (28.8%) | 289 (23.6%) | 10471 (28.6%) |
| Drug overdose | 4604 (13.0%) | 177 (14.4%) | 4781 (13.1%) |
| Head Trauma | 9128 (25.8%) | 421 (34.3%) | 9549 (26.1%) |
| Other | 3577 (10.1%) | 117 (9.5%) | 3694 (10.1%) |
| Missing | 1917 (5.4%) | 82 (6.7%) | 1999 (5.5%) |
| **Recipient HCV Serostatus** |  |  |  |
| Negative | 33366 (94.4%) | 1165 (94.9%) | 34531 (94.5%) |
| Positive | 1400 (4.0%) | 44 (3.6%) | 1444 (4.0%) |
| Missing | 562 (1.6%) | 18 (1.5%) | 580 (1.6%) |
| **Donor Smoking History** |  |  |  |
| No | 26490 (75.0%) | 985 (80.3%) | 27475 (75.2%) |
| Yes | 8129 (23.0%) | 215 (17.5%) | 8344 (22.8%) |
| Missing | 709 (2.0%) | 27 (2.2%) | 736 (2.0%) |
| **Donor Diabetes History** |  |  |  |
| No | 32275 (91.4%) | 1147 (93.5%) | 33422 (91.4%) |
| Yes | 2816 (8.0%) | 73 (5.9%) | 2889 (7.9%) |
| Missing | 237 (0.7%) | 7 (0.6%) | 244 (0.7%) |
| **Donor Hypertension History** |  |  |  |
| No | 24554 (69.5%) | 933 (76.0%) | 25487 (69.7%) |
| Yes | 10501 (29.7%) | 287 (23.4%) | 10788 (29.5%) |
| Missing | 273 (0.8%) | 7 (0.6%) | 280 (0.8%) |
| **Peak Albumin** |  |  |  |
| Median [Q1,Q3] | 3.50 [3.00,3.90] | 3.50 [3.10,3.90] | 3.50 [3.00,3.90] |
| Missing | 1168 (3.3%) | 30 (2.4%) | 1198 (3.3%) |
| **Peak BUN** |  |  |  |
| Median [Q1,Q3] | 22.0 [16.0,31.0] | 23.0 [17.0,32.0] | 22.0 [16.0,31.0] |
| Missing | 233 (0.7%) | 14 (1.1%) | 247 (0.7%) |
| **Peak Na** |  |  |  |
| Median [Q1,Q3] | 149 [144,157] | 150 [145,157] | 149 [144,157] |
| Missing | 65 (0.2%) | 0 (0%) | 65 (0.2%) |
| **Recipient Delayed Graft Function incidence** |  |  |  |
| Yes | 15358 (43.5%) | 356 (29.0%) | 15714 (43.0%) |
| No | 19967(56.5%) | 871 (80.0%) | 20838 (57.0%) |
| Missing | 3 (~0%) | 0 (0%) | 3 (~0%) |
| **Recipient Age (years)** |  |  |  |
| Median [Q1,Q3] | 57.0 [47.0,65.0] | 53.0 [42.0,63.0] | 57.0 [47.0,65.0] |
| **Recipient Sex** |  |  |  |
| Female | 13500 (38.2%) | 496 (40.4%) | 13996 (38.3%) |
| Male | 21828 (61.8%) | 731 (59.6%) | 22559 (61.7%) |
| **Recipient BMI (kg/m²)** |  |  |  |
| Median [Q1,Q3] | 28.5 [24.8,32.6] | 28.3 [24.4,32.6] | 28.5 [24.8,32.6] |
| Missing | 19 (0.1%) | 0 (0%) | 19 (0.1%) |
| **Recipient Ethnicity** |  |  |  |
| White | 13716 (38.8%) | 423 (34.5%) | 14139 (38.7%) |
| Asian | 2681 (7.6%) | 90 (7.3%) | 2771 (7.6%) |
| Black | 11685 (33.1%) | 424 (34.6%) | 12109 (33.1%) |
| Hispanic | 6408 (18.1%) | 265 (21.6%) | 6673 (18.3%) |
| Other | 838 (2.4%) | 25 (2.0%) | 863 (2.4%) |
| **Recipient Primary Diagnosis** |  |  |  |
| Diabetes | 12497 (35.4%) | 367 (29.9%) | 12864 (35.2%) |
| Cystic kidney disease | 3524 (10.0%) | 116 (9.5%) | 3640 (10.0%) |
| Glomerulonephritis | 5104 (14.4%) | 191 (15.6%) | 5295 (14.5%) |
| Hypertension | 8856 (25.1%) | 332 (27.1%) | 9188 (25.1%) |
| Other | 3413 (9.7%) | 131 (10.7%) | 3544 (9.7%) |
| Missing | 1934 (5.5%) | 90 (7.3%) | 2024 (5.5%) |
| **Recipient Diabetes** |  |  |  |
| No | 20408 (57.8%) | 789 (64.3%) | 21197 (58.0%) |
| Yes | 14905 (42.2%) | 437 (35.6%) | 15342 (42.0%) |
| Missing | 15 (0.0%) | 1 (0.1%) | 16 (0.0%) |
| **TRR Serum Creatinine at Time of Transplant** |  |  |  |
| Median [Q1,Q3] | 7.90 [5.74,10.5] | 8.09 [5.89,11.0] | 7.90 [5.74,10.5] |
| Missing | 188 (0.5%) | 3 (0.2%) | 191 (0.5%) |
| **Pre-emptive Transplant** |  |  |  |
| No | 3805 (10.8%) | 134 (10.9%) | 3939 (10.8%) |
| Yes | 31505 (89.2%) | 1092 (89.0%) | 32597 (89.2%) |
| Missing | 18 (0.1%) | 1 (0.1%) | 19 (0.1%) |
| **Recipient Functional Status** |  |  |  |
| ≤60% | 6255 (17.7%) | 192 (15.6%) | 6447 (17.6%) |
| 70% | 9900 (28.0%) | 339 (27.6%) | 10239 (28.0%) |
| 80% | 10354 (29.3%) | 368 (30.0%) | 10722 (29.3%) |
| 90% | 6038 (17.1%) | 223 (18.2%) | 6261 (17.1%) |
| 100% | 1960 (5.5%) | 86 (7.0%) | 2046 (5.6%) |
| Missing | 821 (2.3%) | 19 (1.5%) | 840 (2.3%) |
| **cPRA Category** |  |  |  |
| 0 | 19835 (56.1%) | 518 (42.2%) | 20353 (55.7%) |
| 1-19 | 4235 (12.0%) | 162 (13.2%) | 4397 (12.0%) |
| 20-79 | 5694 (16.1%) | 205 (16.7%) | 5899 (16.1%) |
| 80-89 | 1156 (3.3%) | 53 (4.3%) | 1209 (3.3%) |
| ≥90 | 2296 (6.5%) | 94 (7.7%) | 2390 (6.5%) |
| Missing | 2112 (6.0%) | 195 (15.9%) | 2307 (6.3%) |
| **Induction Agents at Tx** |  |  |  |
| ATG | 22447 (63.5%) | 813 (66.3%) | 23260 (63.6%) |
| ATG + Basiliximab | 1092 (3.1%) | 21 (1.7%) | 1113 (3.0%) |
| Basiliximab without ATG | 4555 (12.9%) | 136 (11.1%) | 4691 (12.8%) |
| Campath | 4448 (12.6%) | 160 (13.0%) | 4608 (12.6%) |
| Other | 1939 (5.5%) | 64 (5.2%) | 2003 (5.5%) |
| Missing | 847 (2.4%) | 33 (2.7%) | 880 (2.4%) |
| **Induction Steroid-Free at Tx** |  |  |  |
| 0 | 10058 (28.5%) | 320 (26.1%) | 10378 (28.4%) |
| 1 | 24895 (70.5%) | 899 (73.3%) | 25794 (70.6%) |
| Missing | 375 (1.1%) | 8 (0.7%) | 383 (1.0%) |
| **Cold Ischemic Time (hours)** |  |  |  |
| Median [Q1, Q3] | 19.5 [15.0,24.0] | 19.6 [15.3,24.0] | 19.5 [15.1,24.0] |
| Missing | 135 (0.4%) | 1 (0.1%) | 136 (0.4%) |
| **HLA Mismatches** |  |  |  |
| ≤2 | 3313 (9.4%) | 109 (8.9%) | 3422 (9.4%) |
| 3 | 5009 (14.2%) | 167 (13.6%) | 5176 (14.2%) |
| 4 | 9996 (28.3%) | 369 (30.1%) | 10365 (28.4%) |
| 5 | 11785 (33.4%) | 404 (32.9%) | 12189 (33.3%) |
| 6 | 5225 (14.8%) | 178 (14.5%) | 5403 (14.8%) |
| **Allocation Type** |  |  |  |
| Local | 23112 (65.4%) | 649 (52.9%) | 23761 (65.0%) |
| Regional | 6095 (17.3%) | 285 (23.2%) | 6380 (17.5%) |
| National | 6121 (17.3%) | 293 (23.9%) | 6414 (17.5%) |
| **Machine perfusion use** |  |  |  |
| SCS | 7163 (20.3%) | 270 (22.0%) | 7433 (20.3%) |
| cHMP | 12324 (34.9%) | 433 (35.3%) | 12757 (34.9%) |
| eiHMP | 2825 (8.0%) | 89 (7.3%) | 2914 (8.0%) |
| toHMP | 6339 (17.9%) | 208 (17.0%) | 6547 (17.9%) |
| Missing | 6677 (18.9%) | 227 (18.5%) | 6904 (18.9%) |
| **Kidney Final Flush Solution** |  |  |  |
| UW | 11023 (31.2%) | 320 (26.1%) | 11343 (31.0%) |
| HTK | 2849 (8.1%) | 72 (5.9%) | 2921 (8.0%) |
| Other | 21445 (60.7%) | 835 (68.1%) | 22280 (60.9%) |
| Missing | 11 (0.0%) | 0 (0%) | 11 (0.0%) |
| **Donor-Recipient Weight Ratio** |  |  |  |
| Median [Q1,Q3] | 1.01 [0.803,1.28] | 1.04 [0.815,1.30] | 1.02 [0.803,1.29] |
| Missing | 261 (0.7%) | 7 (0.6%) | 268 (0.7%) |
| **Time from admission to donation (days)** |  |  |  |
| Median [Q1,Q3] | 5.00 [3.00,8.00] | 6.00 [4.00,9.00] | 5.00 [3.00,8.00] |
| Missing | 156 (0.4%) | 8 (0.7%) | 164 (0.4%) |
| **Transplant Year** |  |  |  |
| Median [Q1,Q3] | 2020 [2020,2020] | 2020 [2020,2020] | 2020 [2020,2020] |
| **Recipient Waitlist Time (Days)** |  |  |  |
| Median [Q1,Q3] | 613 [149,1320] | 470 [96.0,1190] | 608 [147,1310] |
| Missing | 175 (0.5%) | 9 (0.7%) | 184 (0.5%) |

*Supplementary Table 2: Multivariable cox model for 1-year graft survival in the SRR cohort, pooled from 20 imputed datasets. Right-skewed variables not modelled as splines were log2-transformed, so the results relate to change every time the variable doubles. *For restricted cubic splines see Figure 2(A-C) and Supplementary Figure 2. HR= Hazard ratio; TTD = time to death; SBP= Systolic blood pressure; BMI = Body mass index; PDCA = pre-donation cardiac arrest; ATG= Anti-Thymocyte Globulin; HLA= Human Leukocyte Antigen; CHMP= Continuous Hypothermic Machine Perfusion; eHMP= End Hypothermic Machine Perfusion.*

| **Variable** | **Adjusted HR (95% CI)** | **Pvalue** |
| --- | --- | --- |
| **RCS: Donor TTD** | Wald test | 0.469 |
| **RCS: Asystolic time** | Wald test | <0.001 |
| **RCS: Kidney Cold Ischemic Time (Hours)** | Wald test | <0.001 |
| **Donor Gender** |  |  |
| Female | Ref | - |
| Male | 0.992 (0.901 to 1.092) | 0.876 |
| **Donor Ethnicity** |  |  |
| White | Ref | - |
| Asian | 0.959 (0.719 to 1.280) | 0.777 |
| Black | 0.978 (0.849 to 1.126) | 0.756 |
| Hispanic | 1.045 (0.912 to 1.198) | 0.529 |
| Other | 0.732 (0.465 to 1.153) | 0.179 |
| **Deceased Donor-Cause of Death** |  |  |
| Cerebrovascular/stroke | Ref | - |
| Anoxia with PDCA | 0.800 (0.712 to 0.899) | <0.001 |
| Drug overdose | 0.812 (0.697 to 0.946) | 0.008 |
| Head Trauma | 0.799 (0.702 to 0.910) | <0.001 |
| Other | 0.992 (0.859 to 1.145) | 0.910 |
| **Recipient Hepatitis C Status** |  |  |
| Negative | Ref | - |
| Positive | 1.014 (0.837 to 1.227) | 0.889 |
| **Donor Smoking History** |  |  |
| No | Ref | - |
| Yes | 1.059 (0.964 to 1.164) | 0.231 |
| **Deceased Donor-History of Diabetes** |  |  |
| No | Ref | - |
| Yes | 1.279 (1.129 to 1.449) | <0.001 |
| **Deceased Donor-History of Hypertension** |  |  |
| No | Ref | - |
| Yes | 1.187 (1.084 to 1.300) | <0.001 |
| **Recipient Gender** |  |  |
| Female | Ref | - |
| Male | 0.986 (0.893 to 1.090) | 0.790 |
| **Recipient Ethnicity** |  |  |
| White | Ref | - |
| Asian | 0.849 (0.713 to 1.010) | 0.065 |
| Black | 1.026 (0.930 to 1.132) | 0.609 |
| Hispanic | 0.822 (0.725 to 0.933) | 0.002 |
| Other | 0.831 (0.623 to 1.107) | 0.205 |
| **Kidney Recipient Primary Diagnosis @ Transplant** |  |  |
| Diabetes | Ref | - |
| Cystic kidney disease | 0.841 (0.686 to 1.030) | 0.094 |
| Glomerulonephritis | 0.838 (0.694 to 1.012) | 0.066 |
| Hypertension | 0.929 (0.792 to 1.089) | 0.362 |
| Other | 1.151 (0.956 to 1.385) | 0.138 |
| **Recipient Diabetes @ Registration** |  |  |
| No | Ref | - |
| Yes | 1.221 (1.052 to 1.418) | 0.009 |
|  |  |  |
| **Recipient Pretransplant Dialysis @ Transplant** |  |  |
| No | Ref | - |
| Yes | 1.538 (1.321 to 1.790) | <0.001 |
| **Recipient Functional Status @Transplant** |  |  |
| ≤60% | Ref | - |
| 70% | 0.895 (0.801 to 1.001) | 0.053 |
| 80% | 0.864 (0.771 to 0.967) | 0.011 |
| 90% | 0.822 (0.719 to 0.940) | 0.004 |
| 100% | 0.729 (0.589 to 0.902) | 0.004 |
| **Calculated Panel Reactive Antibody** |  |  |
| 0 | Ref | - |
| 1-19 | 1.057 (0.935 to 1.196) | 0.376 |
| 20-79 | 1.094 (0.977 to 1.226) | 0.118 |
| 80-89 | 1.016 (0.797 to 1.294) | 0.900 |
| ≥90 | 1.004 (0.830 to 1.214) | 0.969 |
| **Type Of Induction Immunosuppression** |  |  |
| ATG | Ref | - |
| ATG + Basiliximab | 1.343 (1.105 to 1.633) | 0.003 |
| Basiliximab without ATG | 1.025 (0.907 to 1.159) | 0.692 |
| Campath | 1.087 (0.958 to 1.233) | 0.197 |
| Other | 0.939 (0.775 to 1.139) | 0.524 |
| **Steroids induction at time of transplant** |  |  |
| 0 | Ref | - |
| 1 | 0.891 (0.815 to 0.975) | 0.012 |
| **HLA mismatch level** |  |  |
| ≤2 | Ref | - |
| 3 | 1.108 (0.926 to 1.326) | 0.262 |
| 4 | 1.154 (0.980 to 1.358) | 0.086 |
| 5 | 1.235 (1.051 to 1.451) | 0.010 |
| 6 | 1.308 (1.097 to 1.561) | 0.003 |
| **Allocation type** |  |  |
| Local | Ref | - |
| Regional | 1.067 (0.955 to 1.192) | 0.249 |
| National | 1.177 (1.044 to 1.327) | 0.008 |
| **Machine perfusion use** |  |  |
| SCS | Ref | - |
| cHMP | 0.917 (0.823 to 1.022) | 0.117 |
| eiHMP | 0.851 (0.720 to 1.004) | 0.056 |
| HMP | 0.948 (0.841 to 1.069) | 0.383 |
| **RCS: Donor Age** | Wald test | <0.001 |
| **RCS: Donor BMI** | Wald test | 0.010 |
| **RCS: Donor Peak Albumin** | Wald test | 0.280 |
| **RCS: Donor Peak Creatinine** | Wald test | <0.001 |
| **RCS: Donor Admission-To-Donation Time (Days)** | Wald test | <0.001 |
| **RCS: Age** | Wald test | <0.001 |
| **RCS: Recipient BMI** | Wald test | 0.159 |
| **RCS: Total Days on Kidney Waiting List** | Wald test | 0.888 |
| **RCS: Donor Recipient Weight Ratio** | Wald test | <0.001 |
| **RCS: Transplant Year** | Wald test | <0.001 |

Supplementary Table 3: Multivariable cox model for 1-year patient survival in the SRR cohort, pooled from 20 imputed datasets. Right-skewed variables not modelled as splines were log2-transformed, so the results relate to change every time the variable doubles. *For restricted cubic splines see Figure 2(D-F) and Supplementary Figure 4. HR=Hazard ratio; TTD = time to death; SBP= Systolic blood pressure; BMI = Body mass index; PDCA = pre-donation cardiac arrest; ATG= Anti-Thymocyte Globulin; HLA= Human Leukocyte Antigen; CHMP= Continuous Hypothermic Machine Perfusion; eHMP= End Hypothermic Machine Perfusion.

| **Variable** | **Adjusted HR (95% CI)** | **Pvalue** |
| --- | --- | --- |
| **RCS: Donor TTD** | Wald test | 0.528 |
| **RCS: Asystolic time** | Wald test | 0.441 |
| **RCS: Kidney Cold Ischemic Time (Hours)** | Wald test | 0.317 |
| **Donor Gender** |  |  |
| Female | Ref | - |
| Male | 0.953 (0.838 to 1.085) | 0.469 |
| **Donor Ethnicity** |  |  |
| White | Ref | - |
| Asian | 1.074 (0.733 to 1.573) | 0.713 |
| Black | 1.018 (0.841 to 1.232) | 0.857 |
| Hispanic | 1.234 (1.035 to 1.471) | 0.019 |
| Other | 0.512 (0.243 to 1.079) | 0.078 |
| **Deceased Donor-Cause of Death** |  |  |
| Cerebrovascular/stroke | Ref | - |
| Anoxia with PDCA | 0.846 (0.722 to 0.991) | 0.039 |
| Drug overdose | 0.955 (0.782 to 1.167) | 0.656 |
| Head Trauma | 0.898 (0.755 to 1.067) | 0.221 |
| Other | 1.103 (0.909 to 1.338) | 0.320 |
| **Recipient Hepatitis C Status** |  |  |
| Negative | Ref | - |
| Positive | 0.963 (0.750 to 1.236) | 0.766 |
| **Donor Smoking History** |  |  |
| No | Ref | - |
| Yes | 1.169 (1.034 to 1.321) | 0.012 |
| **Deceased Donor-History of Diabetes** |  |  |
| No | Ref | - |
| Yes | 1.060 (0.888 to 1.265) | 0.522 |
| **Deceased Donor-History of Hypertension** |  |  |
| No | Ref | - |
| Yes | 1.086 (0.961 to 1.227) | 0.185 |
| **Recipient Gender** |  |  |
| Female | Ref | - |
| Male | 1.037 (0.907 to 1.186) | 0.593 |
| **Recipient Ethnicity** |  |  |
| White | Ref | - |
| Asian | 0.729 (0.577 to 0.920) | 0.008 |
| Black | 0.943 (0.828 to 1.075) | 0.380 |
| Hispanic | 0.747 (0.631 to 0.883) | <0.001 |
| Other | 0.873 (0.606 to 1.257) | 0.465 |
| **Kidney Recipient Primary Diagnosis @ Transplant** |  |  |
| Diabetes | Ref | - |
| Cystic kidney disease | 0.778 (0.593 to 1.020) | 0.069 |
| Glomerulonephritis | 0.810 (0.631 to 1.041) | 0.099 |
| Hypertension | 0.868 (0.707 to 1.066) | 0.178 |
| Other | 1.058 (0.832 to 1.347) | 0.644 |
| **Recipient Diabetes @ Registration** |  |  |
| No | Ref | - |
| Yes | 1.533 (1.262 to 1.862) | <0.001 |
| **Recipient Pretransplant Dialysis @ Transplant** |  |  |
| No | Ref | - |
| Yes | 1.698 (1.378 to 2.093) | <0.001 |
| **Recipient Functional Status @Transplant** |  |  |
| ≤60% | Ref | - |
| 70% | 0.920 (0.795 to 1.063) | 0.258 |
| 80% | 0.833 (0.716 to 0.969) | 0.018 |
| 90% | 0.764 (0.636 to 0.918) | 0.004 |
| 100% | 0.721 (0.540 to 0.962) | 0.026 |
| **Calculated Panel Reactive Antibody** |  |  |
| 0 | Ref | - |
| 1-19 | 1.012 (0.856 to 1.196) | 0.892 |
| 20-79 | 1.155 (0.996 to 1.339) | 0.056 |
| 80-89 | 0.907 (0.649 to 1.267) | 0.565 |
| ≥90 | 0.944 (0.728 to 1.223) | 0.661 |
| **Type Of Induction Immunosuppression** |  |  |
| ATG | Ref | - |
| ATG + Basiliximab | 1.459 (1.133 to 1.879) | 0.003 |
| Basiliximab without ATG | 0.959 (0.817 to 1.127) | 0.613 |
| Campath | 1.092 (0.921 to 1.295) | 0.313 |
| Other | 1.056 (0.828 to 1.347) | 0.661 |
| **Steroids induction at time of transplant** |  |  |
| 0 | Ref | - |
| 1 | 0.971 (0.861 to 1.094) | 0.625 |
| **HLA mismatch level** |  |  |
| ≤2 | Ref | - |
| 3 | 1.172 (0.929 to 1.478) | 0.182 |
| 4 | 1.168 (0.943 to 1.446) | 0.154 |
| 5 | 1.149 (0.930 to 1.421) | 0.198 |
| 6 | 1.186 (0.939 to 1.500) | 0.153 |
| **Allocation type** |  |  |
| Local | Ref | - |
| Regional | 1.079 (0.931 to 1.250) | 0.313 |
| National | 1.115 (0.945 to 1.315) | 0.197 |
| **Machine perfusion use** |  |  |
| SCS | Ref | - |
| cHMP | 0.920 (0.793 to 1.068) | 0.273 |
| eiHMP | 0.907 (0.726 to 1.134) | 0.393 |
| HMP | 1.011 (0.860 to 1.188) | 0.893 |
| **RCS: Donor Age** | Wald test | 0.065 |
| **RCS: Donor BMI** | Wald test | 0.819 |
| **RCS: Donor Peak Albumin** | Wald test | 0.040 |
| **RCS: Donor Peak Creatinine** | Wald test | 0.464 |
| **RCS: Donor Admission-To-Donation Time (Days)** | Wald test | 0.303 |
| **RCS: Age** | Wald test | <0.001 |
| **RCS: Recipient BMI** | Wald test | 0.240 |
| **RCS: Total Days on Kidney Waiting List** | Wald test | 0.754 |
| **RCS: Donor Recipient Weight Ratio** | Wald test | 0.732 |
| **RCS: Transplant Year** | Wald test | <0.001 |

Supplementary Table 4: Linear regression model for 1-year eGFR in the SRR cohort, pooled from 20 imputed datasets. Right-skewed variables not modelled as splines were log2-transformed, so the results relate to change every time the variable doubles. *For restricted cubic splines see Supplementary Figure 5. TTD = time to death; SBP= Systolic blood pressure; BMI = Body mass index; PDCA = pre-donation cardiac arrest; ATG= Anti-Thymocyte Globulin; HLA= Human Leukocyte Antigen; CHMP= Continous Hypothermic Machine Perfusion; eHMP= End Hypothermic Machine Perfusion.

| Variable | Coef(95%CI) | Pvalue |
| --- | --- | --- |
| **RCS: Donor TTD** | Wald test | 0.393 |
| **RCS: Asystolic time** | Wald test | <0.001 |
| **RCS: Kidney Cold Ischemic Time (Hours)** | Wald test | <0.001 |
| **Donor Gender** |  |  |
| Female | Ref | - |
| Male | -0.277 (-0.814 to 0.260) | 0.311 |
| **Donor Ethnicity** |  |  |
| White | Ref | - |
| Asian | -2.499 (-4.142 to -0.856) | 0.003 |
| Black | -0.412 (-1.229 to 0.405) | 0.323 |
| Hispanic | 1.504 (0.726 to 2.283) | <0.001 |
| Other | 3.072 (1.037 to 5.106) | 0.003 |
| **Deceased Donor-Cause of Death** |  |  |
| Cerebrovascular/stroke | Ref | - |
| Anoxia with PDCA | 3.528 (2.834 to 4.221) | <0.001 |
| Drug overdose | 4.780 (3.922 to 5.637) | <0.001 |
| Head Trauma | 3.286 (2.540 to 4.032) | <0.001 |
| Other | 2.221 (1.341 to 3.100) | <0.001 |
| **Recipient Hepatitis C Status** |  |  |
| Negative | Ref | - |
| Positive | 0.163 (-0.957 to 1.284) | 0.775 |
| **Donor Smoking History** |  |  |
| No | Ref | - |
| Yes | 0.186 (-0.373 to 0.746) | 0.514 |
| **Deceased Donor-History of Diabetes** |  |  |
| No | Ref | - |
| Yes | -4.021 (-4.857 to -3.186) | <0.001 |
| **Deceased Donor-History of Hypertension** |  |  |
| No | Ref | - |
| Yes | -2.626 (-3.171 to -2.080) | <0.001 |
| **Recipient Gender** |  |  |
| Female | Ref | - |
| Male | 3.758 (3.206 to 4.310) | <0.001 |
| **Recipient Ethnicity** |  |  |
| White | Ref | - |
| Asian | 4.267 (3.353 to 5.181) | <0.001 |
| Black | -6.157 (-6.725 to -5.590) | <0.001 |
| Hispanic | 3.754 (3.082 to 4.426) | <0.001 |
| Other | 0.868 (-0.611 to 2.347) | 0.250 |
| **Kidney Recipient Primary Diagnosis @ Transplant** |  |  |
| Diabetes | Ref | - |
| Cystic kidney disease | -0.287 (-1.414 to 0.840) | 0.618 |
| Glomerulonephritis | -1.008 (-2.063 to 0.048) | 0.061 |
| Hypertension | 0.018 (-0.931 to 0.968) | 0.970 |
| Other | -2.344 (-3.454 to -1.234) | <0.001 |
| **Recipient Diabetes @ Registration** |  |  |
| No | Ref | - |
| Yes | 0.161 (-0.724 to 1.046) | 0.722 |
| **Recipient Pretransplant Dialysis @ Transplant** |  |  |
| No | Ref | - |
| Yes | -0.421 (-1.152 to 0.310) | 0.259 |
| **Recipient Functional Status @Transplant** |  |  |
| ≤60% | Ref | - |
| 70% | 0.516 (-0.146 to 1.178) | 0.127 |
| 80% | 0.672 (0.005 to 1.339) | 0.048 |
| 90% | 0.678 (-0.068 to 1.423) | 0.075 |
| 100% | 1.441 (0.365 to 2.518) | 0.009 |
| **Calculated Panel Reactive Antibody** |  |  |
| 0 | Ref | - |
| 1-19 | -0.034 (-0.736 to 0.667) | 0.924 |
| 20-79 | -0.274 (-0.907 to 0.360) | 0.398 |
| 80-89 | -0.309 (-1.620 to 1.001) | 0.644 |
| ≥90 | -0.433 (-1.419 to 0.553) | 0.389 |
| **Type Of Induction Immunosuppression** |  |  |
| ATG | Ref | - |
| ATG + Basiliximab | 0.820 (-0.497 to 2.136) | 0.222 |
| Basiliximab without ATG | 1.002 (0.318 to 1.686) | 0.004 |
| Campath | -0.652 (-1.334 to 0.030) | 0.061 |
| Other | 1.123 (0.114 to 2.131) | 0.029 |
| **Steroids induction at time of transplant** |  |  |
| 0 | Ref | - |
| 1 | -0.115 (-0.617 to 0.387) | 0.653 |
| **HLA mismatch level** |  |  |
| ≤2 | Ref | - |
| 3 | -0.246 (-1.177 to 0.685) | 0.605 |
| 4 | -0.418 (-1.265 to 0.430) | 0.334 |
| 5 | -0.610 (-1.450 to 0.229) | 0.154 |
| 6 | -1.148 (-2.096 to -0.200) | 0.018 |
| **Allocation type** |  |  |
| Local | Ref | - |
| Regional | -0.670 (-1.312 to -0.028) | 0.041 |
| National | -1.788 (-2.492 to -1.084) | <0.001 |
| **Machine perfusion use** |  |  |
| SCS | Ref | - |
| cHMP | 0.596 (-0.021 to 1.213) | 0.058 |
| eiHMP | 1.131 (0.174 to 2.088) | 0.021 |
| HMP | 0.745 (0.057 to 1.433) | 0.034 |
| **RCS: Donor Age** | Wald test | <0.001 |
| **RCS: Donor BMI** | Wald test | <0.001 |
| **RCS: Donor Peak Albumin** | Wald test | 0.073 |
| **RCS: Donor Peak Creatinine** | Wald test | <0.001 |
| **RCS: Donor Admission-To-Donation Time (Days)** | Wald test | <0.001 |
| **RCS: Age** | Wald test | <0.001 |
| **RCS: Recipient BMI** | Wald test | <0.001 |
| **RCS: Total Days on Kidney Waiting List** | Wald test | 0.047 |
| **RCS: Donor Recipient Weight Ratio** | Wald test | <0.001 |
| **RCS: Transplant Year** | Wald test | <0.001 |

Supplementary Table 5: Logistic regression model for Delayed graft function incidence in the SRR cohort, pooled from 20 imputed datasets. Right-skewed variables not modelled as splines were log2-transformed, so the results relate to change every time the variable doubles. *For restricted cubic splines see Figure 2(G-I) and Supplementary Figure 6. OR = Odds ratio; TTD = time to death; SBP= Systolic blood pressure; BMI = Body mass index; PDCA = pre-donation cardiac arrest; ATG= Anti-Thymocyte Globulin; HLA= Human Leukocyte Antigen; CHMP= Continuous Hypothermic Machine Perfusion; eHMP= End Hypothermic Machine Perfusion.

| **Variable** | **Adjusted OR (95% CI)** | **Pvalue** |
| --- | --- | --- |
| **RCS: Donor TTD** | Wald test | 0.039 |
| **RCS: Asystolic time** | Wald test | <0.001 |
| **RCS: Kidney Cold Ischemic Time (Hours)** | Wald test | <0.001 |
| **Donor Gender** |  |  |
| Female | Ref | - |
| Male | 1.356 (1.279 to 1.436) | <0.001 |
| **Donor Ethnicity** |  |  |
| White | Ref | - |
| Asian | 0.960 (0.807 to 1.141) | 0.642 |
| Black | 0.893 (0.819 to 0.974) | 0.010 |
| Hispanic | 0.915 (0.843 to 0.992) | 0.032 |
| Other | 0.936 (0.753 to 1.163) | 0.552 |
| **Deceased Donor-Cause of Death** |  |  |
| Cerebrovascular/stroke | Ref | - |
| Anoxia with PDCA | 0.772 (0.716 to 0.831) | <0.001 |
| Drug overdose | 0.721 (0.657 to 0.792) | <0.001 |
| Head Trauma | 0.869 (0.804 to 0.940) | <0.001 |
| Other | 0.802 (0.730 to 0.881) | <0.001 |
| **Recipient Hepatitis C Status** |  |  |
| Negative | Ref | - |
| Positive | 1.170 (1.041 to 1.315) | 0.009 |
| **Donor Smoking History** |  |  |
| No | Ref | - |
| Yes | 1.032 (0.973 to 1.094) | 0.292 |
| **Deceased Donor-History of Diabetes** |  |  |
| No | Ref | - |
| Yes | 1.009 (0.924 to 1.102) | 0.842 |
| **Deceased Donor-History of Hypertension** |  |  |
| No | Ref | - |
| Yes | 1.099 (1.038 to 1.164) | 0.001 |
| **Recipient Gender** |  |  |
| Female | Ref | - |
| Male | 1.526 (1.438 to 1.619) | <0.001 |
| **Recipient Ethnicity** |  |  |
| White | Ref | - |
| Asian | 1.536 (1.395 to 1.692) | <0.001 |
| Black | 1.601 (1.508 to 1.700) | <0.001 |
| Hispanic | 1.534 (1.429 to 1.647) | <0.001 |
| Other | 1.471 (1.261 to 1.717) | <0.001 |
| **Kidney Recipient Primary Diagnosis @ Transplant** |  |  |
| Diabetes | Ref | - |
| Cystic kidney disease | 0.875 (0.776 to 0.986) | 0.028 |
| Glomerulonephritis | 0.810 (0.725 to 0.906) | <0.001 |
| Hypertension | 0.870 (0.788 to 0.962) | 0.006 |
| Other | 0.909 (0.809 to 1.021) | 0.107 |
| **Recipient Diabetes @ Registration** |  |  |
| No | Ref | - |
| Yes | 1.117 (1.017 to 1.227) | 0.020 |
| **Recipient Pretransplant Dialysis @ Transplant** |  |  |
| No | Ref | - |
| Yes | 6.696 (6.000 to 7.473) | <0.001 |
| **Recipient Functional Status @Transplant** |  |  |
| ≤60% | Ref | - |
| 70% | 0.939 (0.877 to 1.006) | 0.074 |
| 80% | 0.857 (0.799 to 0.918) | <0.001 |
| 90% | 0.763 (0.704 to 0.826) | <0.001 |
| 100% | 0.678 (0.604 to 0.761) | <0.001 |
| **Calculated Panel Reactive Antibody** |  |  |
| 0 | Ref | - |
| 1-19 | 0.971 (0.901 to 1.047) | 0.446 |
| 20-79 | 0.982 (0.917 to 1.052) | 0.603 |
| 80-89 | 0.863 (0.751 to 0.993) | 0.040 |
| ≥90 | 0.912 (0.821 to 1.014) | 0.090 |
| **Type Of Induction Immunosuppression** |  |  |
| ATG | Ref | - |
| ATG + Basiliximab | 1.171 (1.022 to 1.342) | 0.023 |
| Basiliximab without ATG | 1.047 (0.974 to 1.125) | 0.216 |
| Campath | 1.101 (1.025 to 1.183) | 0.008 |
| Other | 0.680 (0.609 to 0.758) | <0.001 |
| **Steroids induction at time of transplant** |  |  |
| 0 | Ref | - |
| 1 | 0.979 (0.928 to 1.033) | 0.434 |
| **HLA mismatch level** |  |  |
| ≤2 | Ref | - |
| 3 | 1.064 (0.962 to 1.177) | 0.228 |
| 4 | 1.138 (1.038 to 1.247) | 0.006 |
| 5 | 1.143 (1.043 to 1.253) | 0.004 |
| 6 | 1.197 (1.081 to 1.326) | <0.001 |
| **Allocation type** |  |  |
| Local | Ref | - |
| Regional | 1.082 (1.011 to 1.158) | 0.022 |
| National | 1.129 (1.047 to 1.217) | 0.002 |
| **Machine perfusion use** |  |  |
| SCS | Ref | - |
| cHMP | 0.475 (0.445 to 0.508) | <0.001 |
| eiHMP | 0.450 (0.407 to 0.498) | <0.001 |
| HMP | 0.552 (0.512 to 0.595) | <0.001 |
| **RCS: Donor Age** | Wald test | <0.001 |
| **RCS: Donor BMI** | Wald test | <0.001 |
| **RCS: Donor Peak Albumin** | Wald test | 0.179 |
| **RCS: Donor Peak Creatinine** | Wald test | <0.001 |
| **RCS: Donor Admission-To-Donation Time (Days)** | Wald test | <0.001 |
| **RCS: Age** | Wald test | 0.185 |
| **RCS: Recipient BMI** | Wald test | <0.001 |
| **RCS: Total Days on Kidney Waiting List** | Wald test | <0.001 |
| **RCS: Donor Recipient Weight Ratio** | Wald test | <0.001 |
| **RCS: Transplant Year** | Wald test | <0.001 |

Supplementary Table 6: Multivariable cox model for 5-year graft survival in the SRR cohort, pooled from 20 imputed datasets. Right-skewed variables not modelled as splines were log2-transformed, so the results relate to change every time the variable doubles. *For restricted cubic splines see Supplementary Figure 7. HR= Hazard ratio; TTD = time to death; SBP= Systolic blood pressure; PDCA = pre-donation cardiac arrest; ATG= Anti-Thymocyte Globulin; HLA= Human Leukocyte Antigen; SCS= Static cold storage and CHMP= Continuous Hypothermic Machine Perfusion; eHMP= End Hypothermic Machine Perfusion.

| **Variable** | **Adjusted HR (95% CI)** | **Pvalue** |
| --- | --- | --- |
| **RCS: Donor TTD** | Wald test | 0.179 |
| **RCS: Asystolic time** | Wald test | 0.074 |
| **RCS: Kidney Cold Ischemic Time (Hours)** | Wald test | <0.001 |
| **Donor Gender** |  |  |
| Female | Ref | - |
| Male | 0.995 (0.939 to 1.054) | 0.871 |
| **Donor Ethnicity** |  |  |
| White | Ref | - |
| Asian | 1.093 (0.924 to 1.295) | 0.300 |
| Black | 1.120 (1.030 to 1.217) | 0.008 |
| Hispanic | 0.965 (0.885 to 1.051) | 0.412 |
| Other | 0.870 (0.676 to 1.119) | 0.277 |
| **Deceased Donor-Cause of Death** |  |  |
| Cerebrovascular/stroke | Ref | - |
| Anoxia with PDCA | 0.863 (0.804 to 0.927) | <0.001 |
| Drug overdose | 0.881 (0.802 to 0.967) | 0.008 |
| Head Trauma | 0.880 (0.814 to 0.951) | 0.001 |
| Other | 0.961 (0.878 to 1.051) | 0.384 |
| **Recipient Hepatitis C Status** |  |  |
| Negative | Ref | - |
| Positive | 1.087 (0.972 to 1.215) | 0.145 |
| **Donor Smoking History** |  |  |
| No | Ref | - |
| Yes | 1.002 (0.945 to 1.062) | 0.951 |
| **Deceased Donor-History of Diabetes** |  |  |
| No | Ref | - |
| Yes | 1.270 (1.173 to 1.376) | <0.001 |
| **Deceased Donor-History of Hypertension** |  |  |
| No | Ref | - |
| Yes | 1.158 (1.095 to 1.225) | <0.001 |
| **Recipient Gender** |  |  |
| Female | Ref | - |
| Male | 1.053 (0.992 to 1.119) | 0.090 |
| **Recipient Ethnicity** |  |  |
| White | Ref | - |
| Asian | 0.689 (0.617 to 0.770) | <0.001 |
| Black | 1.038 (0.979 to 1.101) | 0.215 |
| Hispanic | 0.831 (0.770 to 0.896) | <0.001 |
| Other | 1.008 (0.862 to 1.179) | 0.922 |
| **Kidney Recipient Primary Diagnosis @ Transplant** |  |  |
| Diabetes | Ref | - |
| Cystic kidney disease | 0.766 (0.676 to 0.867) | <0.001 |
| Glomerulonephritis | 0.826 (0.738 to 0.924) | <0.001 |
| Hypertension | 0.962 (0.874 to 1.058) | 0.421 |
| Other | 0.999 (0.893 to 1.118) | 0.990 |
| **Recipient Diabetes @ Registration** |  |  |
| No | Ref | - |
| Yes | 1.237 (1.131 to 1.354) | <0.001 |
| **Recipient Pretransplant Dialysis @ Transplant** |  |  |
| No | Ref | - |
| Yes | 1.526 (1.392 to 1.673) | <0.001 |
| **Recipient Functional Status @Transplant** |  |  |
| ≤60% | Ref | - |
| 70% | 0.931 (0.871 to 0.997) | 0.039 |
| 80% | 0.877 (0.818 to 0.941) | <0.001 |
| 90% | 0.885 (0.816 to 0.959) | 0.003 |
| 100% | 0.768 (0.679 to 0.868) | <0.001 |
| **Calculated Panel Reactive Antibody** |  |  |
| 0 | Ref | - |
| 1-19 | 1.102 (1.024 to 1.187) | 0.010 |
| 20-79 | 1.139 (1.064 to 1.218) | <0.001 |
| 80-89 | 1.119 (0.972 to 1.287) | 0.117 |
| ≥90 | 1.189 (1.066 to 1.326) | 0.002 |
| **Type Of Induction Immunosuppression** |  |  |
| ATG | Ref | - |
| ATG + Basiliximab | 1.143 (1.010 to 1.295) | 0.035 |
| Basiliximab without ATG | 1.084 (1.010 to 1.164) | 0.026 |
| Campath | 1.070 (0.995 to 1.152) | 0.068 |
| Other | 0.990 (0.887 to 1.104) | 0.854 |
| **Steroids induction at time of transplant** |  |  |
| 0 | Ref | - |
| 1 | 0.990 (0.938 to 1.046) | 0.729 |
| **HLA mismatch level** |  |  |
| ≤2 | Ref | - |
| 3 | 1.102 (0.993 to 1.223) | 0.068 |
| 4 | 1.101 (1.000 to 1.211) | 0.049 |
| 5 | 1.182 (1.076 to 1.299) | <0.001 |
| 6 | 1.199 (1.080 to 1.331) | <0.001 |
| **Allocation type** |  |  |
| Local | Ref | - |
| Regional | 1.000 (0.932 to 1.072) | 0.989 |
| National | 1.098 (1.016 to 1.187) | 0.019 |
| **Machine perfusion use** |  |  |
| SCS | Ref | - |
| cHMP | 0.914 (0.856 to 0.975) | 0.007 |
| eiHMP | 0.833 (0.749 to 0.925) | <0.001 |
| HMP | 0.924 (0.858 to 0.996) | 0.039 |
| **RCS: Donor Age** | Wald test | <0.001 |
| **RCS: Donor BMI** | Wald test | <0.001 |
| **RCS: Donor Peak Albumin** | Wald test | 0.099 |
| **RCS: Donor Peak Creatinine** | Wald test | 0.014 |
| **RCS: Donor Admission-To-Donation Time (Days)** | Wald test | 0.002 |
| **RCS: Age** | Wald test | <0.001 |
| **RCS: Recipient BMI** | Wald test | 0.028 |
| **RCS: Total Days on Kidney Waiting List** | Wald test | 0.383 |
| **RCS: Donor Recipient Weight Ratio** | Wald test | <0.001 |
| **RCS: Transplant Year** | Wald test | <0.001 |

*Supplementary Table 7: Multivariable cox model for 5-year patient survival in the SRR cohort, pooled from 20 imputed datasets. Right-skewed variables not modelled as splines were log2-transformed, so the results relate to change every time the variable doubles. *For restricted cubic splines see Supplementary Figure 8. HR= Hazard ratio; TTD = time to death; SBP= Systolic blood pressure; PDCA = pre-donation cardiac arrest; ATG= Anti-Thymocyte Globulin; HLA= Human Leukocyte Antigen; SCS= Static cold storage and CHMP= Continous Hypothermic Machine Perfusion; eHMP= End Hypothermic Machine Perfusion.*

| **Variable** | **Adjusted HR (95% CI)** | **Pvalue** |
| --- | --- | --- |
| **RCS: Donor TTD** | Wald test | 0.277 |
| **RCS: Asystolic time** | Wald test | 0.091 |
| **RCS: Kidney Cold Ischemic Time (Hours)** | Wald test | 0.017 |
| **Donor Gender** |  |  |
| Female | Ref | - |
| Male | 0.971 (0.906 to 1.041) | 0.403 |
| **Donor Ethnicity** |  |  |
| White | Ref | - |
| Asian | 1.082 (0.880 to 1.329) | 0.455 |
| Black | 1.127 (1.019 to 1.246) | 0.020 |
| Hispanic | 0.998 (0.900 to 1.107) | 0.971 |
| Other | 0.788 (0.569 to 1.092) | 0.152 |
| **Deceased Donor-Cause of Death** |  |  |
| Cerebrovascular/stroke | Ref | - |
| Anoxia with PDCA | 0.889 (0.816 to 0.969) | 0.008 |
| Drug overdose | 0.948 (0.849 to 1.060) | 0.352 |
| Head Trauma | 0.916 (0.835 to 1.004) | 0.062 |
| Other | 0.993 (0.891 to 1.106) | 0.896 |
| **Recipient Hepatitis C Status** |  |  |
| Negative | Ref | - |
| Positive | 1.117 (0.982 to 1.270) | 0.093 |
| **Donor Smoking History** |  |  |
| No | Ref | - |
| Yes | 1.027 (0.959 to 1.099) | 0.446 |
| **Deceased Donor-History of Diabetes** |  |  |
| No | Ref | - |
| Yes | 1.133 (1.028 to 1.249) | 0.012 |
| **Deceased Donor-History of Hypertension** |  |  |
| No | Ref | - |
| Yes | 1.085 (1.015 to 1.160) | 0.016 |
| **Recipient Gender** |  |  |
| Female | Ref | - |
| Male | 1.106 (1.029 to 1.190) | 0.007 |
| **Recipient Ethnicity** |  |  |
| White | Ref | - |
| Asian | 0.603 (0.527 to 0.688) | <0.001 |
| Black | 0.866 (0.806 to 0.929) | <0.001 |
| Hispanic | 0.778 (0.711 to 0.851) | <0.001 |
| Other | 0.899 (0.742 to 1.089) | 0.277 |
| **Kidney Recipient Primary Diagnosis @ Transplant** |  |  |
| Diabetes | Ref | - |
| Cystic kidney disease | 0.671 (0.578 to 0.779) | <0.001 |
| Glomerulonephritis | 0.730 (0.636 to 0.837) | <0.001 |
| Hypertension | 0.872 (0.780 to 0.975) | 0.016 |
| Other | 0.920 (0.805 to 1.053) | 0.226 |
| **Recipient Diabetes @ Registration** |  |  |
| No | Ref | - |
| Yes | 1.380 (1.241 to 1.535) | <0.001 |
| **Recipient Pretransplant Dialysis @ Transplant** |  |  |
| No | Ref | - |
| Yes | 1.621 (1.451 to 1.811) | <0.001 |
| **Recipient Functional Status @Transplant** |  |  |
| ≤60% | Ref | - |
| 70% | 0.974 (0.900 to 1.054) | 0.510 |
| 80% | 0.858 (0.789 to 0.932) | <0.001 |
| 90% | 0.824 (0.747 to 0.909) | <0.001 |
| 100% | 0.663 (0.566 to 0.778) | <0.001 |
| **Calculated Panel Reactive Antibody** |  |  |
| 0 | Ref | - |
| 1-19 | 1.083 (0.991 to 1.183) | 0.077 |
| 20-79 | 1.116 (1.028 to 1.211) | 0.009 |
| 80-89 | 1.069 (0.899 to 1.272) | 0.448 |
| ≥90 | 1.106 (0.964 to 1.268) | 0.151 |
| **Type Of Induction Immunosuppression** |  |  |
| ATG | Ref | - |
| ATG + Basiliximab | 1.238 (1.072 to 1.429) | 0.004 |
| Basiliximab without ATG | 1.066 (0.982 to 1.158) | 0.126 |
| Campath | 1.079 (0.986 to 1.181) | 0.098 |
| Other | 1.053 (0.926 to 1.198) | 0.428 |
| **Steroids induction at time of transplant** |  |  |
| 0 | Ref | - |
| 1 | 1.004 (0.941 to 1.072) | 0.901 |
| **HLA mismatch level** |  |  |
| ≤2 | Ref | - |
| 3 | 1.141 (1.009 to 1.291) | 0.035 |
| 4 | 1.084 (0.967 to 1.214) | 0.167 |
| 5 | 1.173 (1.048 to 1.312) | 0.005 |
| 6 | 1.121 (0.988 to 1.270) | 0.075 |
| **Allocation type** |  |  |
| Local | Ref | - |
| Regional | 0.977 (0.898 to 1.063) | 0.594 |
| National | 1.022 (0.929 to 1.125) | 0.655 |
| **Machine perfusion use** |  |  |
| SCS | Ref | - |
| cHMP | 0.906 (0.836 to 0.981) | 0.015 |
| eiHMP | 0.852 (0.750 to 0.968) | 0.014 |
| HMP | 0.965 (0.884 to 1.054) | 0.430 |
| **RCS: Donor Age** | Wald test | <0.001 |
| **RCS: Donor BMI** | Wald test | 0.342 |
| **RCS: Donor Peak Albumin** | Wald test | 0.339 |
| **RCS: Donor Peak Creatinine** | Wald test | 0.423 |
| **RCS: Donor Admission-To-Donation Time (Days)** | Wald test | 0.261 |
| **RCS: Age** | Wald test | <0.001 |
| **RCS: Recipient BMI** | Wald test | 0.024 |
| **RCS: Total Days on Kidney Waiting List** | Wald test | 0.012 |
| **RCS: Donor Recipient Weight Ratio** | Wald test | 0.012 |
| **RCS: Transplant Year** | Wald test | <0.001 |

Supplementary Table 8: Multivariable cox model for impact of FTTD on 1-year graft survival in the SRR cohort, pooled from 20 imputed datasets. Right-skewed variables not modelled as splines were log2-transformed, so the results relate to change every time the variable doubles. *For restricted cubic splines see Supplementary Figure 9. HR= Hazard ratio; TTD = time to death; SBP= Systolic blood pressure; BMI = Body mass index; PDCA = pre-donation cardiac arrest; ATG= Anti-Thymocyte Globulin; HLA= Human Leukocyte Antigen; SCS= Static cold storage and CHMP= Continuous Hypothermic Machine Perfusion; eHMP= End Hypothermic Machine Perfusion.

| **Variable** | **Adjusted HR (95% CI)** | **Pvalue** |
| --- | --- | --- |
| **RCS: Donor FTTD** | Wald test | 0.248 |
| **RCS: Asystolic time** | Wald test | <0.001 |
| **RCS: Kidney Cold Ischemic Time (Hours)** | Wald test | <0.001 |
| **Donor Gender** |  |  |
| Female | Ref | - |
| Male | 0.992 (0.901 to 1.092) | 0.868 |
| **Donor Ethnicity** |  |  |
| White | Ref | - |
| Asian | 0.963 (0.722 to 1.285) | 0.799 |
| Black | 0.980 (0.851 to 1.129) | 0.781 |
| Hispanic | 1.046 (0.913 to 1.199) | 0.515 |
| Other | 0.736 (0.467 to 1.159) | 0.186 |
| **Deceased Donor-Cause of Death** |  |  |
| Cerebrovascular/stroke | Ref | - |
| Anoxia with PDCA | 0.801 (0.713 to 0.899) | <0.001 |
| Drug overdose | 0.812 (0.697 to 0.946) | 0.007 |
| Head Trauma | 0.798 (0.701 to 0.908) | <0.001 |
| Other | 0.993 (0.861 to 1.146) | 0.928 |
| **Recipient Hepatitis C Status** |  |  |
| Negative | Ref | - |
| Positive | 1.013 (0.837 to 1.227) | 0.892 |
| **Donor Smoking History** |  |  |
| No | Ref | - |
| Yes | 1.058 (0.964 to 1.163) | 0.236 |
| **Deceased Donor-History of Diabetes** |  |  |
| No | Ref | - |
| Yes | 1.281 (1.131 to 1.451) | <0.001 |
| **Deceased Donor-History of Hypertension** |  |  |
| No | Ref | - |
| Yes | 1.185 (1.082 to 1.298) | <0.001 |
| **Recipient Gender** |  |  |
| Female | Ref | - |
| Male | 0.986 (0.892 to 1.090) | 0.785 |
| **Recipient Ethnicity** |  |  |
| White | Ref | - |
| Asian | 0.848 (0.712 to 1.010) | 0.064 |
| Black | 1.026 (0.930 to 1.132) | 0.610 |
| Hispanic | 0.822 (0.725 to 0.933) | 0.002 |
| Other | 0.829 (0.622 to 1.105) | 0.202 |
| **Kidney Recipient Primary Diagnosis @ Transplant** |  |  |
| Diabetes | Ref | - |
| Cystic kidney disease | 0.842 (0.688 to 1.032) | 0.098 |
| Glomerulonephritis | 0.839 (0.695 to 1.013) | 0.068 |
| Hypertension | 0.929 (0.792 to 1.089) | 0.363 |
| Other | 1.152 (0.957 to 1.386) | 0.136 |
| **Recipient Diabetes @ Registration** |  |  |
| No | Ref | - |
| Yes | 1.222 (1.053 to 1.419) | 0.008 |
| **Recipient Pretransplant Dialysis @ Transplant** |  |  |
| No | Ref | - |
| Yes | 1.540 (1.322 to 1.793) | <0.001 |
| **Recipient Functional Status @Transplant** |  |  |
| ≤60% | Ref | - |
| 70% | 0.894 (0.800 to 1.000) | 0.050 |
| 80% | 0.862 (0.770 to 0.966) | 0.011 |
| 90% | 0.821 (0.718 to 0.938) | 0.004 |
| 100% | 0.727 (0.587 to 0.899) | 0.003 |
| **Calculated Panel Reactive Antibody** |  |  |
| 0 | Ref | - |
| 1-19 | 1.059 (0.936 to 1.198) | 0.367 |
| 20-79 | 1.095 (0.978 to 1.226) | 0.116 |
| 80-89 | 1.016 (0.797 to 1.295) | 0.897 |
| ≥90 | 1.005 (0.831 to 1.215) | 0.962 |
| **Type Of Induction Immunosuppression** |  |  |
| ATG | Ref | - |
| ATG + Basiliximab | 1.346 (1.108 to 1.637) | 0.003 |
| Basiliximab without ATG | 1.025 (0.906 to 1.159) | 0.696 |
| Campath | 1.084 (0.955 to 1.230) | 0.211 |
| Other | 0.941 (0.776 to 1.141) | 0.535 |
| **Steroids induction at time of transplant** |  |  |
| 0 | Ref | - |
| 1 | 0.892 (0.816 to 0.976) | 0.013 |
| **HLA mismatch level** |  |  |
| ≤2 | Ref | - |
| 3 | 1.109 (0.927 to 1.327) | 0.259 |
| 4 | 1.153 (0.979 to 1.357) | 0.088 |
| 5 | 1.233 (1.050 to 1.449) | 0.011 |
| 6 | 1.307 (1.096 to 1.559) | 0.003 |
| **Allocation type** |  |  |
| Local | Ref | - |
| Regional | 1.066 (0.955 to 1.191) | 0.255 |
| National | 1.174 (1.041 to 1.324) | 0.009 |
| **Machine perfusion use** |  |  |
| SCS | Ref | - |
| cHMP | 0.916 (0.822 to 1.021) | 0.113 |
| eiHMP | 0.850 (0.720 to 1.003) | 0.055 |
| HMP | 0.947 (0.840 to 1.067) | 0.372 |
| **RCS: Donor Age** | Wald test | <0.001 |
| **RCS: Donor BMI** | Wald test | 0.008 |
| **RCS: Donor Peak Albumin** | Wald test | 0.271 |
| **RCS: Donor Peak Creatinine** | Wald test | <0.001 |
| **RCS: Donor Admission-To-Donation Time (Days)** | Wald test | <0.001 |
| **RCS: Age** | Wald test | <0.001 |
| **RCS: Recipient BMI** | Wald test | 0.158 |
| **RCS: Total Days on Kidney Waiting List** | Wald test | 0.884 |
| **RCS: Donor Recipient Weight Ratio** | Wald test | <0.001 |
| **RCS: Transplant Year** | Wald test | <0.001 |
